# Synthesis, Structures and Properties of Cu(II) and Mn(II) Complexes with 1,10-Phenanthroline-2-carboxylic acid and 2,2’-Bipyridine Ligands

**DOI:** 10.3390/molecules15118349

**Published:** 2010-11-15

**Authors:** Jingya Sun, Huanzhi Xu

**Affiliations:** College of Marine Sciences, Zhejiang Ocean University, Zhoushan 316000, China; E-Mail: huanzhixu2009@163.com (H.X.)

**Keywords:** π···π interactions, ion exchange, crystal structure

## Abstract

Four new 2,2’-bipyridine and 1,10-phenanthroline complexes, namely [Mn(phenca)_2_]·(H_2_O)_2_ (**1**), [Cu_4_(phen)_4_(OH^-^)_4_(H_2_O)_2_](DMF)_4_(ClO_4_^-^)_4_(H_2_O) (**2**), [Cu_2_(2,2-bipy)_2_(C_2_O_4_^2-^)(H_2_O)_2_(NO_3_)_2_] (**3**) and [Cu(2,2-bipy)_2_(ClO_4_^-^)](ClO_4_^-^) (**4**) (2,2’-bpy = 2,2’-bipyridine, Hphenca = 1,10-phenanthroline-2-carboxylic acid) have been synthesized by a hydrothermal reaction. The products were characterized by elemental analysis, infrared spectroscopy and X-ray crystal diffraction. While strong hydrogen bonds play central roles in the formation of the 3D structure, the combined influence of the weak interactions such as π···π interactions is also evident in the structures. A preliminary investigation on the ion exchange properties of the complexes is presented.

## 1. Introduction

Recently, interest in supramolecular chemistry and crystal engineering has been focused on the design strategies and construction of architectures with diversity topologies [1]. One of the most effective strategies is to select organic building blocks with diverse connection modes as directing nodes to assemble multidimensional frameworks by binding metal centers [2].

Self-assembly is the fundamental molecular recognition process adopted by Nature to generate the elegant and intricate molecular machinery from which life is built. Various weak dispersive interactions, such as hydrogen bonds, π···π stacking, hydrophobic charge-transfer, electrostatic as well as metal ion coordination, represent the backbone of self-assembly processes and supramolecular architectures [3]. Aromatic π···π stacking interactions between the π-electron clouds of aromatic systems have been extensively observed in many areas of chemistry and biochemistry [4,5,6,7,8,9,10]. They play an essential role in the structures of biological macromolecules such as the double helical structure of DNA, the tertiary structure of proteins and porphyrin aggregation, and are exploited for intercalation of drugs into DNA [11,12,13].

Owing to the strong functionality of π···π stacking interactions, a number of experimental and theoretical methods have been employed to investigate the nature of aromatic-aromatic interaction [14]. Likewise, experimental proofs for carbonyl···π interactions, and more generally for lone pair···π interactions, are presented in the literature [15,16]. Moreover, some molecular architectures, such as helices, cyclophanes and host-guest assemblies, have also been formed through π stacking interactions [17,18]. In this context, we have succeeded in obtaining four novel complexes [Mn(phenca)_2_] (H_2_O)_2_ (**1**)_, _[Cu_4_(phen)_4_(OH^-^)_4_(H_2_O)_2_](DMF)_4_(ClO_4_^-^)**_4_**(H_2_O) (**2**), [Cu_2_(2,2-bipy)_2_(C_2_O_4_^2-^)(H_2_O)_2_(NO_3_)_2_] (**3**) and [Cu (2,2-bipy)_2_(ClO_4_^-^**)**](ClO_4_^-^**)** (**4**) based on 2,2’-biprydine and 1,10-phenanthroline-2-carboxylic acid ligands. Strong hydrogen bonds and π···π interactions play important roles in the formation of their 3D structures.

## 2. Results and Discussion

### 2.1. Crystal structures of compounds ***1**-**4***

**Figure 1:**
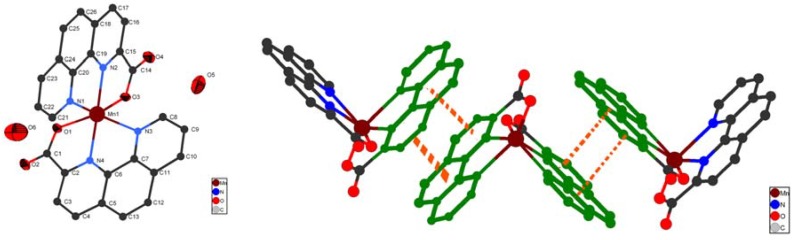
**(a)** The coordination environments of the Mn atom of complex **1** with 30% thermal ellipsolids. **(b)** A perspective view of the π-π stacking interaction between pyridine rings. All the hydrogen atoms are omitted for clarity.

Single-crystal X-ray diffraction measurement reveals that the crystal structure of complex **1** conforms to the space group P-1. A molecular structure showing the arrangement about the Mn (II) metal center is shown in Figure 1(a). The Mn(II) ion is six-coordinated and adopts a distorted octahedron coordination geometry by coordinating to four nitrogen atoms from two phenca ligands (Mn-N(1) 2.440 Å; Mn -N(2) 2.177Å; Mn -N(3) 2.369 Å; Mn -N(4) 2.183 Å) and two oxygen atoms from carboxyl group of the phenca ligand (Mn -O(1) 2.160Å; Mn -O(3) 2.156Å) with N1 and O3 in the axial positions.

**Figure 2:**
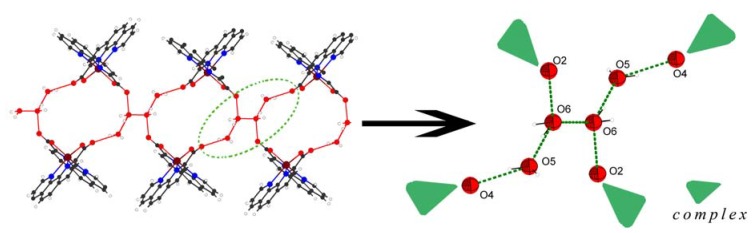
Perspective view of (H_2_O)_4_ morphology in compound **1**. Packing diagram of the supramolecular edifice viewed along the crystallographic b axis, all the hydrogen atoms are omitted for clarity.

As illustrated in Figure 1(b), stacking interactions play an important role in the formation of the 2D structure [Figure 2(b)]. The adjacent complexes are further connected through face-to-face π-π stacking between pyridine rings (with centroid to centroid distance of 3.644–3.689Å, and an angle of 109°–113°). This interaction is similar to that observed in other recently reported compounds [19,20].

It must be remarked that two lattice water molecules and their symmetric equivalents form a (H_2_O)_4_ water cluster and the structure is stabilized by strong hydrogen bonds linking the vertex of the water cluster (Figure 2). All of them are hydrogen bonded through an oxygen atom of a COO group (O4 or O2).

The detailed structure of the water cluster is shown in Figure 2. Carboxyl groups provide potential hydrogen bonding intermolecular interactions, beside its coordination ability towards metal ions. As shown in Figure 2, the O6 adopt triangle distorted geometries with double hydrogen-bond donors, while O5 display single hydrogen-bond donor and acceptor. Within the water cluster, the O···O distances are in range of 2.65(1)–2.88(8) Å with an average of 2.78(5) Å, which is comparable to that observed in the ice II phase 2.77–2.84 Å [21].

**Figure 3:**
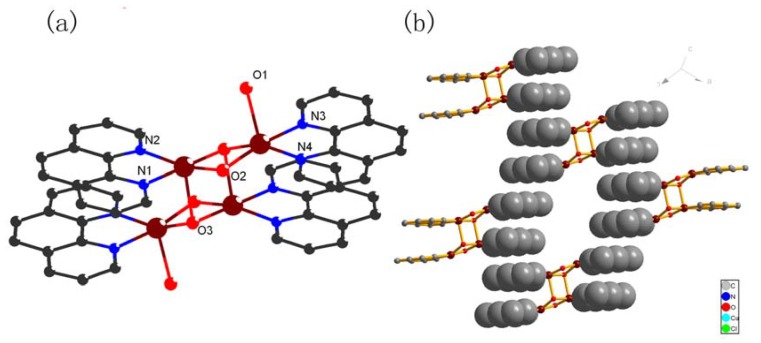
**(a)** The coordination environment of the Cu atoms of complex **2** with 30% thermal ellipsolids. **(b)** The π···π stacking interactions shown in a space filling model.

Complex **2** conforms to the space group P-1 (Table 1). A molecular structure showing the arrangement about the Cu (II) metal center is shown in Figure 3(a). The Cu atom is five-coordinated and adopts a distorted tetrahedron coordination geometry by coordinating to two nitrogen atoms from the phen ligands and three oxygen atoms from water molecules, with O1 or O3 in the vertex position.

**Table 1:** Crystal data and structure refinements for complexes **1–4**.

Complexes	1	2		3	4
Empirical formula	C_26_H_18_MnN_4_O_6_	C_60_H_72_Cl_4_Cu_4_N_12_O_28_		C_22_H_20_Cu_2_N_6_O_12_	C_20_H_16_Cl_2_CuN_4_O_8_
Formula mass	537.38	1801.22		687.52	574.81
Temperature(K)	296(2) K	296(2) K		296(2) K	296(2) K
Crystal system	Triclinic	Triclinic		Monoclinic	Triclinic
Space group	p-1	p-1		P2(1)/n	P-1
a(Å)	10.0889(9)	10.1983(18)		7.7035(2)	7.4088(19)
b(Å)	10.5636(9)	14.267(2)		10.2045(3)	11.227(3)
c(Å)	12.1510(10)	15.162(4)		16.5347(5)	14.877(4)
α(°)	71.456(3)	114.261(12)		90	110.164(8)
β(°)	68.256(3)	92.692(11)	100.132(2)		96.489(9)
γ(°)	82.130(4)	110.063(8)	90		99.608(8)
V(Å^3^)	1140.13(17)	1843.2(6)	1279.53(6)		1125.7(5)
Z	2	1	2		2
D_calc_(gcm^-3^)	1.565	1.623	1.784		1.696
μ(mm^−1^)	0.632	1.373	1.740		1.264
F[000]	550		696		582
θ(°)	1.89 to 25.00	1.51 to 25.01	2.36 to 25.01		1.98 to 25.01
Data/restraints/parameters	3943 /0 / 334	920	2259 / 0 / 190		3915 / 0 / 308
Goodness-of-fit on F^2^	1.029	1.000	1.096		1.080
Final R^a^ indices[I>2σ(I)]	R1 = 0.0624, wR2 = 0.1658	R1 = 0.0627, wR2 = 0.1734	R1 = 0.0267, wR2 = 0.0752		R1 = 0.0777, wR2 = 0.2403
R(int)	0.0457	0.0931	0.0266		0.0272
R indices (all data)	R1 = 0.1232, wR2 = 0.1963	R1 = 0.1358, wR2 = 0.1979	R1 = 0.0325, wR2 = 0.0786		R1 = 0.0883, wR2 = 0.2552

^a^ R_ 1_= Σ(|F_0_|-|F_c_|)/Σ|F_0_|; wR_2_=[Σw(|F_0_^2^|-|F_c_^2^)^2^/Σw(|F_0_^2^|^2^)]^1/2^

These complexes are extended through π···π stacking between the phen forming a 2D network (with central to central distance of 3.61–3.63Å). There are 1D channels along the b-axis, and the perchlorate anions are filled into the channels through strong hydrogen bonds as shown in Figure 3(b). It is noteworthy that the perchlorate anion is not coordinated to the 3D framework due to its weak coordination ability.

**Figure 4:**
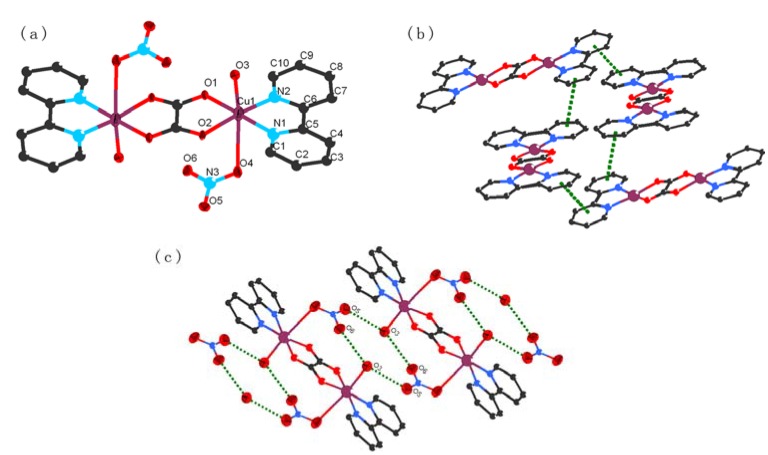
**(a)** The coordination environment of the centeral metal ions of complex **3** with 50% thermal ellipsoids. **(b)** A perspective view of the π-π stacking interaction between 2,2’-pyridine rings. **(c)** Packing diagram connected by hydrogen bonding. All hydrogen atoms are omitted for clarity.

An ORTEP view of the molecular structure of complex **3** is depicted in Figure 4(a). The selected molecular geometry parameters are listed in Table 2. The Cu(II) ion is six-coordinated and adopts z distorted octahedron coordination geometry by coordinating to two nitrogen atoms from two 2,2’-bipyridine ligands (Cu-N(1) 1.969Å; Cu-N(2) 1.984Å) and four oxygen atoms from an oxalic acid molecule, a coordinated water molecule and nitrate ion (Cu-O(1) 1.9700 Å; Cu-O(2) 1.9864 Å; Cu-O(3) 2.344 Å; Cu-O(4) 2.761Å) with O3 and O4 in the axial positions.

**Table 2:** Selected bond lengths (Å) and angles (°) for complexes **1-4**.

Complex 1			
Mn(1)-O(3)	2.156(4)	Mn(1)-N(2)	2.177(4)
Mn(1)-O(1)	2.160(4)	Mn(1)-N(4)	2.183(4)
Mn(1)-N(3)	2.370(5)	Mn(1)-N(1)	2.440(4)
O(3)-Mn(1)-O(1)	105.32(16)	O(3)-Mn(1)-N(2)	73.80(15)
O(1)-Mn(1)-N(2)	118.99(16)	O(3)-Mn(1)-N(4)	127.70(15)
O(1)-Mn(1)-N(4)	73.27(17)	N(2)-Mn(1)-N(4)	153.89(17)
O(3)-Mn(1)-N(3)	92.17(15)	O(1)-Mn(1)-N(3)	143.85(16)
N(2)-Mn(1)-N(3)	96.02(16)	N(4)-Mn(1)-N(3)	71.02(16)
O(3)-Mn(1)-N(1)	143.97(15)	O(1)-Mn(1)-N(1)	92.31(15)
N(2)-Mn(1)-N(1)	70.16(16)	N(4)-Mn(1)-N(1)	87.16(16)
N(3)-Mn(1)-N(1)	91.40(15)		
Complex 2			
Cu(1)-O(2)	1.919(4)	Cu(1)-O(3)#1	1.971(4)
Cu(1)-N(1)	2.023(5)	Cu(1)-N(2)	2.035(4)
Cu(1)-O(3)	2.335(4)	Cu(1)-Cu(2)	2.9347(9)
Cu(2)-O(2)	1.937(4)	Cu(2)-O(3)#1	1.973(3)
Cu(2)-N(4)	2.016(5)	Cu(2)-N(3)	2.030(5)
Cu(2)-O(1)	2.250(5)	O(3)-Cu(1)#1	1.971(4)
O(3)-Cu(2)#1	1.973(3)	O(2)-Cu(1)-O(3)#1	81.55(15)
O(2)-Cu(1)-N(1)	96.60(17)	O(3)#1-Cu(1)-N(1)	178.04(16)
O(2)-Cu(1)-N(2)	164.89(18)	O(3)#1-Cu(1)-N(2)	100.31(17)
N(1)-Cu(1)-N(2)	81.30(18)	O(2)-Cu(1)-O(3)	97.81(16)
O(3)#1-Cu(1)-O(3)	86.15(15)	N(1)-Cu(1)-O(3)	94.79(17)
N(2)-Cu(1)-O(3)	97.28(15)	O(2)-Cu(1)-Cu(2)	40.68(11)
O(2)-Cu(2)-O(3)#1	81.05(15)	O(2)-Cu(2)-N(4)	95.43(19)
O(3)#1-Cu(2)-N(4)	156.91(19)	O(2)-Cu(2)-N(3)	171.42(19)
O(3)#1-Cu(2)-N(3)	98.89(18)	N(4)-Cu(2)-N(3)	81.2(2)
O(2)-Cu(2)-O(1)	96.55(18)	O(3)#1-Cu(2)-O(1)	101.24(17)
N(4)-Cu(2)-O(1)	101.83(19)	N(3)-Cu(2)-O(1)	91.88(19)
Symmetry transformations used to generate equivalent atoms: #1 -x+1,-y+1,-z+1			
Complex 3			
Cu(1)-O(1)	2.0135(18)	N(1)-Cu(1)-N(4)	97.27(10)
Cu(1)-N(3)	2.018(2)	O(1)-Cu(1)-N(2)	93.09(8)
Cu(1)-N(1)	2.036(2)	N(3)-Cu(1)-N(2)	98.90(9)
Cu(1)-N(4)	2.041(2)	N(1)-Cu(1)-N(2)	77.45(9)
Cu(1)-O(1)	2.0135(18)	N(4)-Cu(1)-N(2)	99.83(9)
Cu(1)-O(2)#1	2.325(2)	O(1)-Cu(1)-O(2)#1	76.85(7)
O(1)-Cu(1)-N(3)	92.97(9)	N(3)-Cu(1)-O(2)#1	88.32(9)
O(1)-Cu(1)-N(1)	90.37(8)	N(1)-Cu(1)-O(2)#1	95.82(9)
N(3)-Cu(1)-N(1)	175.19(8)	N(4)-Cu(1)-O(2)#1	90.83(8)
O(1)-Cu(1)-N(4)	166.14(9)	N(2)-Cu(1)-O(2)#1	167.98(8)
N(3)-Cu(1)-N(4)	80.18(10)		
Symmetry transformations used to generate equivalent atoms: #1 -x+1,-y+2,-z+1			
Complex 4			
Cu(1)-N(3)	1.973(6)	N(1)-Cu(1)-N(4)	103.5(2)
Cu(1)-N(1)	1.984(5)	N(2)-Cu(1)-N(4)	151.9(2)
Cu(1)-N(2)	1.994(5)	N(3)-Cu(1)-N(2)	102.2(2)
Cu(1)-N(4)	1.995(6)	N(1)-Cu(1)-N(2)	81.7(2)
N(3)-Cu(1)-N(1)	160.9(2)	N(3)-Cu(1)-N(4)	81.9(3)

In the crystal structure of **3**, the oxalic acid acts as the bridge ligand which links two copper atoms, while two 2,2’-bipy molecules act as terminal ligands on each side. Analysis of the crystal packing of the title compound reveals the existence of multiple intermolecular π-π stacking interaction (with an average centroid-to-centroid distance of 3.923Å) between the 2,2’-bipyridine molecules, forming a two-dimensional sheet [Figure 4(b)]. In addition, O-H-O hydrogen bonds play important roles in the formation of the 2D structure, in which O3 acts as double hydrogen-bond donors towards O5 and O6 of the nitrate ion [Figure 4(c)].

**Figure 5:**
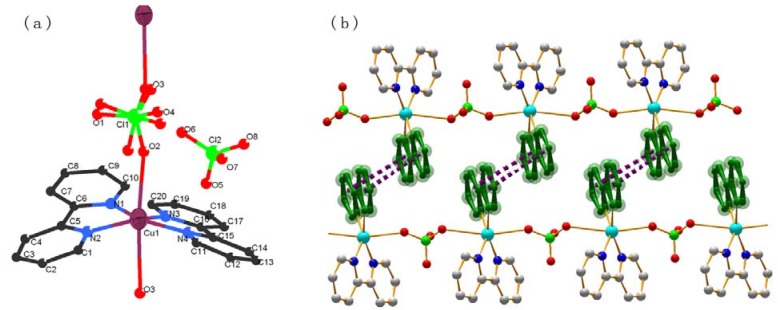
**(a)** The coordination environment of the Cu atoms of complex **4** with 30% thermal ellipsolids. **(b)** A perspective view of the π-π stacking interaction between pyridine rings. All the hydrogen atoms are omitted for clarity.

Single-crystal X-ray diffraction measurement reveals that the crystals of complex **4** conform to the space group P-1. A molecular structure showing the arrangement about the Cu (II) mental center is shown in Figure 5(a). The structure consists of a polymeric three-dimensional network in which each metal atom is six-coordinated and adopts a distorted octahedron coordination geometry by coordinating to four nitrogen atoms from two 2,2’-bipyridine molecules (Cu-N(1) 1.984Å; Cu-N(2) 1.994Å; Cu-N(3) 1.973Å; Cu-N(4) 1.995Å) lie on the equatorial plane and two oxygen atoms from the perchlorate anion Cu-O(2) 2.490 Å; Cu-O(2) 2.742 Å) in the axial positions. The structure is expanded into a chain along a axis by using the perchlorate anion as a bridge ligand. The chains are further extended by the π-π stacking interactions between the adjacent 2,2’-bipyridine molecules to form a 3D structure. In this structure, the terminal pyridyl rings are overlapped with a centroid-to-centroid distance of 3.62 to 3.96 Å.

**Figure 6:**
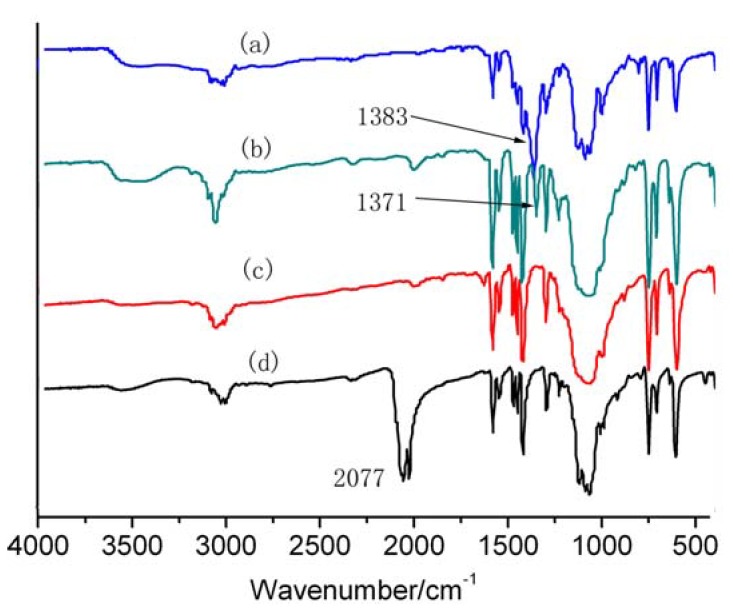
FT-IR spectra of **(a)** complex **4** treated with aqueous solution of NaNO_3_, **(b)** complex **4** treated with aqueous solution of NaNO_2_, **(c)** complex **4**, **(d)** complex **4** treated with aqueous solution of KSCN.

As revealed by the crystal structure analysis of complex **4**, the perchlorate anions are located within the open structure of the 1D channel through C-H-O hydrogen bond and π-π stacking interactions (Figure 5). Complex **4** is expected to display anion exchange properties since it is insoluble in common solvents. The FT-IR and XRD spectra of the exchanged product and the original **4** are shown in Figure 6 and Table S6, respectively. Characteristic intense bands appear about 1,383 cm^−1^, which originate from the NO_3_^-^ ion [22], while the characteristic intense bands in the range from 2,077 to 2,130 cm^−1^ originate from the SCN^-^ ion [23,24]. Furthermore, the elemental analysis result also indicated anion exchange, but the exchange was incomplete.

TGA (Figure s1- s4) were carried out in the interest of studying the thermal behavior of complexes **1-4**. We selected complex **1** as an example to study the thermal behavior. The TGA indicates that **1** loses 6.7% of total weight in the 30–90 °C temperature range, corresponding to the removal of two lattice water molecules per formula unit (calcd. 6.7%). When the temperature continues rising, the products lose 83% of the total weight in the 200–300 °C temperature range, corresponding to the removal of the phenca ligand (Figure s1). Complexes **2-4** also display good thermal stability.

## 3. Experimental

### 3.1. Materials and Physical Measurements

All chemicals were commercial materials of analytical grade and used without further purification. FT-IR spectra were recorded on a Nicolet Magna-IR 550 spectrometer in dry KBr pellets. C, H and N analysis was measured on a MOD 1106 elemental analyzer. The PXRD data were collected on a Bruker D8 diffractometer with Cu Kα radiation (λ = 1.5418Å).

### 3.2. Synthesis of ***1-4***

#### 3.2.1. Synthesis of [Mn(phenca)_2_](H_2_O)_2_ (**1**)

A solution of phenca (0.224 g, 1 mmol) and Mn(CH_3_COO)_2_ (0.172 g, 1 mmol) in water (10 mL) was stirred for 30 min at room temperature. Slow evaporation of the solvent at room temperature led to the formation of yellow block crystals of **1**. Yield: 68% (based on Mn). Anal. Calcd for C_26_H_18_MnN_4_O_6_ (%): C, 58.11; H, 3.38; N, 10.43. Found: C, 58.12; H, 3.37; N, 10.41. IR (cm^−1^): 3,300, 1,685 m (v C=O), 1,585 (v C=N) 1,510, 1,305 s, 849 s.

#### 3.2.2. Synthesis of [Cu_4_(phen)_4_(OH-)_4_(H_2_O)_2_](DMF)_4_(ClO_4_-)_4_(H_2_O) (**2**)

A solution of phen (0.18 g, 1 mmol) and Cu(ClO_4_)_2_ (0.152 g, 1 mmol) in DMF/H_2_O (10 mL, 1:1) was stirred for 10 min at room temperature and placed in a 25 mL Teflon-lined autoclave and heated at 150 °C for 120 h. The autoclave was cooled over a period of 8 h at 10 °C h^−1^, and compound **2**, obtained as blue block crystals, was collected by filtration, and dried at ambient temperature. Yield: 59% (based on Cu). Anal. Calcd for C_60_H_72_Cl_4_Cu_4_N_12_O_28_ (3): C, 40.01; H, 3.81; N, 9.33. Found (%): C, 40.79; H, 4.02; N, 9.25. IR (cm^−1^): 3,420, 1,695m (v C=O), 1,575 (v C=N), 1,110.

#### 3.2.3. Synthesis of [Cu_2_(2,2-bipy)_2_(C_2_O_4_^2-^)(H_2_O)_2_(NO_3_)_2_] (**3**)

A solution of 2,2’-bipyridine (0.312 g, 2 mmol) and Cu(NO_3_)_2_ (0.241 g, 1 mmol) in DMF (10 mL) was slowly added to a solution of oxalic acid (0.180 g, 2mmol) in DMF (10 mL). The mixture was stirred for 10 min at 298 K and then placed in a 25 mL Teflon-lined autoclave and heated at 150 °C for 120 h. The autoclave was cooled over a period of 8 h at 10 °C h^−1^, and **3** was collected as blue block crystals by filtration, and dried at ambient temperature. Yield: 60% (based on Cu). Anal. Calcd for C_22_H_20_Cl_2_Cu_2_N_6_O_1__2_ (1): C, 48.29; H, 3.90; N, 11.60. Found (%): C,48.65; H, 3.91; N, 11.82. IR (cm^−1^): 3,320, 1,692 m (v C=O), 1,563 (v C=N), 1,380, 856.

#### 3.2.4. Synthesis of [Cu (2,2-bipy)_2_(ClO_4-_)](ClO_4-_) (**4**)

A solution of 2,2’-bipyridine (0.156 g, 1 mmol) and Cu(ClO_4_)_2_ (0.076 g, 0.5 mmol) in ethanol (10 mL) was stirred for 10 min at room temperature and then placed in a 25 mL Teflon-lined autoclave and heated at 150 °C for 120 h. The autoclave was cooled over a period of 8 h at 10 °C h^−1^, and **4** was collected by filtration as blue block crystals, that were dried at ambient temperature. Yield: 60% (based on Cu). Anal. Calcd for C_20_H_16_Cl_2_CuN_4_O_8_ (3): C, 41.29; H, 2.80; N, 9.60. Found (%): C, 41.79; H, 2.81; N, 9.75. IR (cm^−1^): 1,670m (v C=O), 1,480, 1,110, 656.

### 3.3. Single-crystal Structure Determination

Intensity data for 1-4 were collected at 296 K on a Bruker SMART CCD area detector diffractometer using graphite-monochromated Mo-Kα radiation (λ = 0.71073 Å) using the *ω-θ* scan mode in the range 1.51 ≤ θ ≤ 25.01°. Raw frame data were integrated with the SAINT [25] program. The structure was solved by direct methods using SHELXS-97 and refined by full-matrix least-squares on F^2^ using SHELXS-97 [26]. An empirical absorption correction was applied with SADABS [26]. All non-hydrogen atoms were refined anisotropically. Hydrogen atoms were set in calculated positions and refined by a riding mode, with a common thermal parameter. All calculations and graphics were performed with SHELXTL [25] and DIAMOND. The crystallographic data and experimental details for the structure analysis are summarized in Table 1.

## 4. Conclusions

In conclusion, we have prepared and characterized four complexes: [Mn(phenca)_2_](H_2_O)_2_ (**1**)*,* [Cu_4_(phen)_4_(OH^-^)_4_(H_2_O)_2_](DMF)_4_(ClO_4_^-^)_4_(H_2_O) (**2**), [Cu_2_(2,2-bipy)_2_(C_2_O_4_^2-^)(H_2_O)_2_(NO_3_)_2_] (**3**) and [Cu(2,2-bipy)_2_(ClO_4_^-^)](ClO_4_^-^) (**4**) and determined their crystal structures. Hydrogen bonding, π···π stacking interactions and cation···π interactions are found to form the packing structure. The complexes also display strong thermal stability.

## Supplementary Materials

Supplementary materials can be found at http://www.mdpi.com/1420-3049/15/11/8349/s1.
