# Supplementary material for: Synthesis, Structures and Properties of Cu(II) and Mn(II) Complexes with 1,10-Phenanthroline-2-carboxylic acid and 2,2’-Bipyridine Ligands
Source: Molecules. 2010 Nov 15;15(11):8349–59. doi: 10.3390/molecules15118349 (PMC6259098; doi:10.3390/molecules15118349)

Synthesis, structures and properties of Cu(II) and Mn(II) complexes with 1,10-phenanthroline-2-carboxylic acid and 2,2'-bipyridine ligand

Jingya Sun, Huanzhi Xu

College of Marine Sciences, Zhejiang Ocean University, Zhoushan 316000, PR China

Figure s1. TG-DTG curve of complex 1.

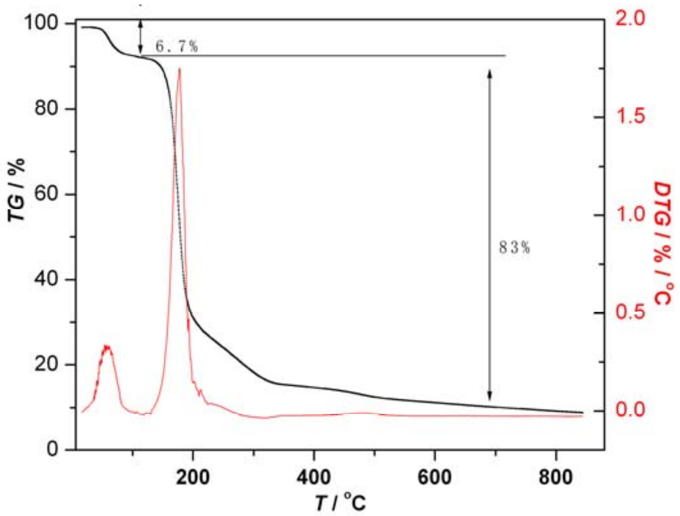

Figure S2. TG-DTG of curve of complex 2.

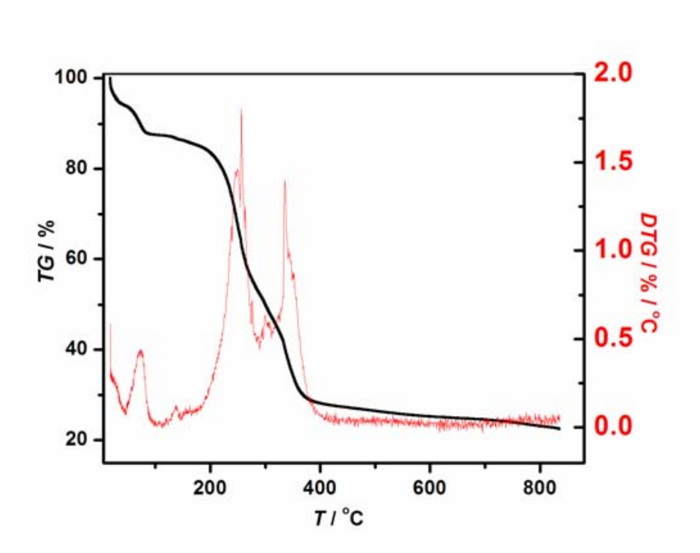

Figure S3. TG-DTG of curve of complex 3.

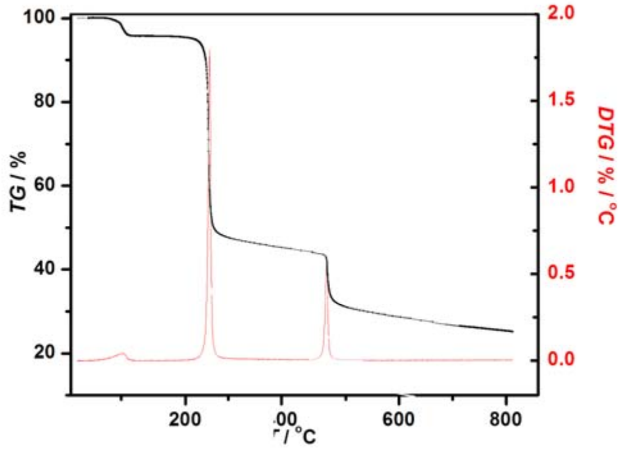

Figure S4. TG-DTG of curve of complex 4.

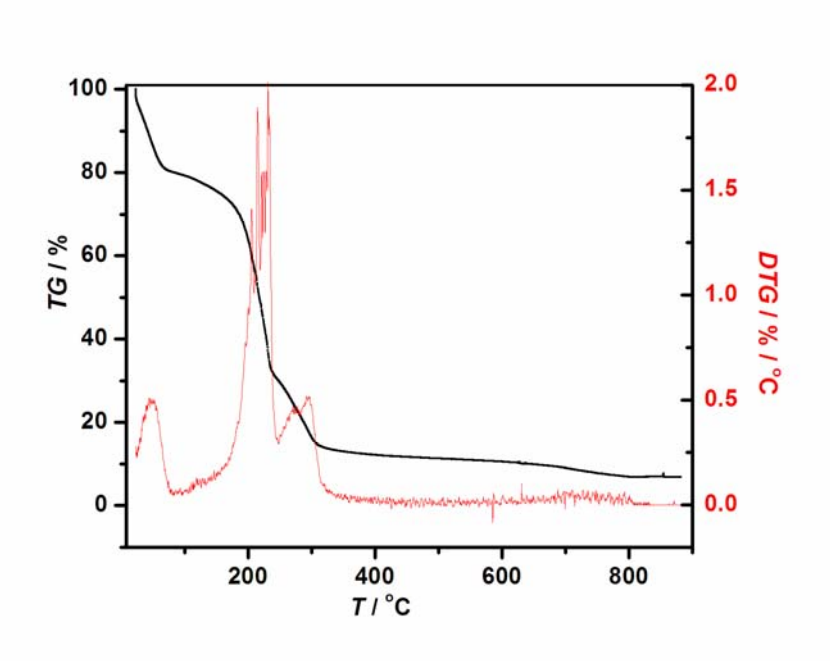

Figure s5. Projection of the crystal packing of polymorph 4 on the bc plane with perchlorate ion encapsulated within the channel.

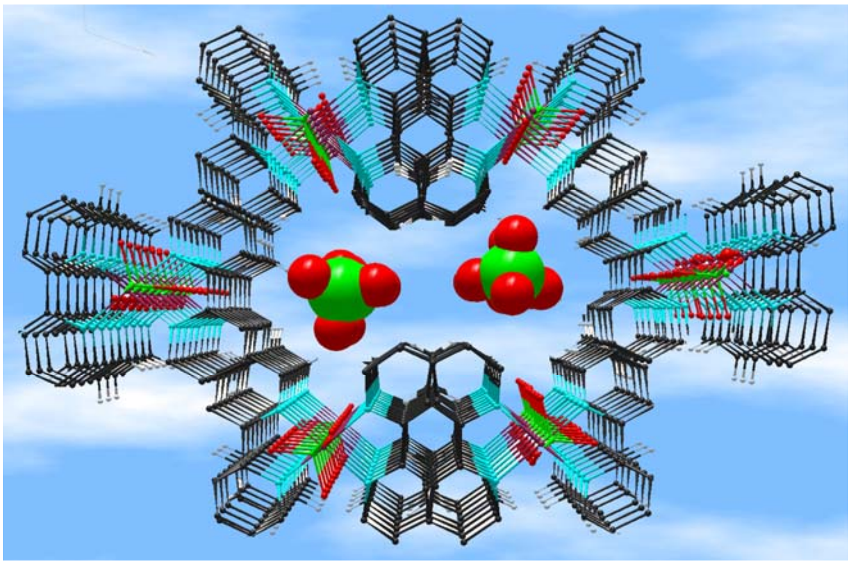

Figure s6. XRD pattern of (a) complex 4, (b) complex 4 treated with aqueous solution of $NaNO_2$, (c) complex 4 treated with aqueous solution of KSCN, (d) complex 4 treated with aqueous solution of $NaNO_3$.

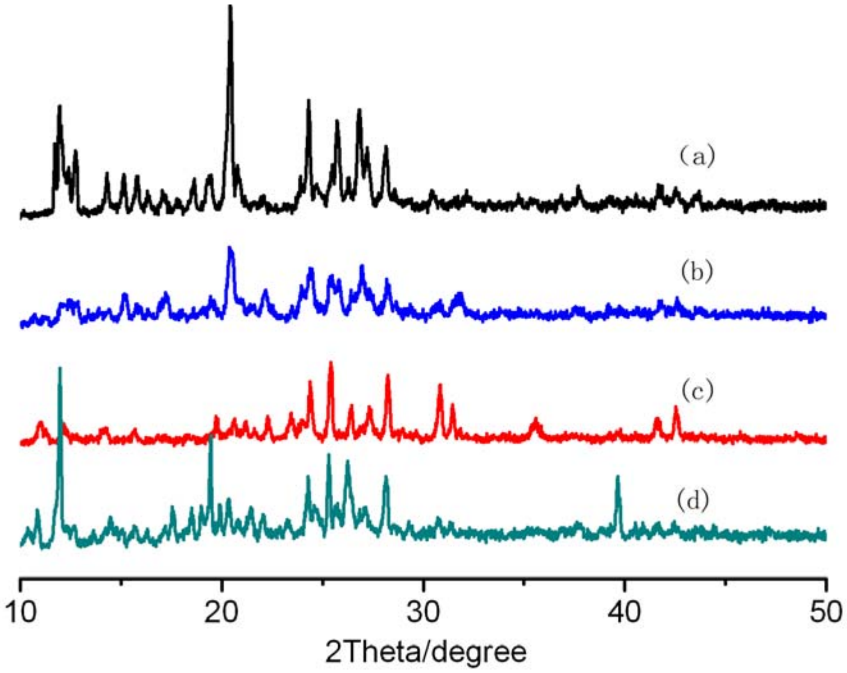

Supplement: Supplementary file 1 [file molecules-15-08349-s001.pdf]
